# HepGentox: a novel promising HepG2 reportergene-assay for the detection of genotoxic substances in complex mixtures

**DOI:** 10.7717/peerj.11883

**Published:** 2021-07-27

**Authors:** Elisabeth Pinter, Christina Friedl, Alexandra Irnesberger, Thomas Czerny, Tina Piwonka, Alfonso Peñarroya, Manfred Tacker, Elisabeth Riegel

**Affiliations:** Departement of Applied Life Sciences, University of Applied Sciences Vienna, FH Campus Wien, Vienna, Austria

**Keywords:** Genotoxicity, Complex Mixtures, Reportergene-assay, p53, Food Contact Materials, HepG2, Metabolization

## Abstract

**Background:**

In risk assessment, genotoxicity is a key factor to determine the safety for the consumer. Most *in vitro* genotoxicity assays were developed for the assessment of pure substances. However, in recent years more attention has been given to complex mixtures, where usually low amounts of a substance are present. For high-throughput screening, a toxicologically sensitive assay should be used, covering a broad range of genotoxic substances and detecting them at low concentrations. HepG2 cells have been recommended as one of the prime candidates for genotoxicity testing, as they are p53 competent, less prone towards cytotoxic effects and tend to have some metabolic activity.

**Methods:**

A HepG2 liver cell line was characterized for its suitability for genotoxicity assessment. For this, a luciferase based reporter gene assay revolving around the p53 pathway was validated for the analysis of pure substances and of complex mixtures. Further, the cell’s capability to detect genotoxins correctly with and without an exogenous metabolizing system, namely rat liver S9, was assessed.

**Results:**

The assay proved to have a high toxicological sensitivity (87.5%) and specificity (94%). Further, the endogenous metabolizing system of the HepG2 cells was able to detect some genotoxins, which are known to depend on an enzymatic system. When complex mixtures were added this did not lead to any adverse effects concerning the assays performance and cytotoxicity was not an issue.

**Discussion:**

The HepGentox proved to have a high toxicological sensitivity and specificity for the tested substances, with similar or even lower lowest effective concentration (LEC) values, compared to other regulatory mammalian assays. This combines some important aspects in one test system, while also being less time and material consuming and covering several genotoxicity endpoints. As the assay performs well with and without an exogenous metabolizing system, no animal liver fractions have to be used, which application is discussed controversially and is considered to be expensive and laborious in sample testing. Because of this, the HepGentox is suitable for a cost-efficient first screening approach to obtain important information with human cells for further approaches, with a relatively fast and easy method. Therefore, the HepGentox is a promising assay to detect genotoxic substances correctly in complex mixtures even at low concentrations, with the potential for a high throughput application. In a nutshell, as part of an *in vitro* bioassay test battery, this assay could provide valuable information for complex mixtures.

## Introduction

Genotoxicity covers a broad term, as it includes any kind of alteration to the DNA, such as mutations, but also changes in the cell cycle and or interactions with cell proliferation. In mammalian cells, several pathways are involved in regulating the response to genotoxic substances, such as the mTOR, the MGMT, the MMR and the p53 pathway (Feng et al., 2005; Klapacz et al., 2016). Genotoxicity testing is an important aspect to gain toxicological information and the OECD guideline for genotoxicity testing (OECD, 2015) has established a variety of tests, which can be applied. These usually include well established assays, such as the bacteria reverse mutation test, the micronucleus test, the mouse lymphoma assay, the chromosomal aberration test, the comet assay and the sister chromatid exchange test. Those assays mainly focus on one genotoxicity endpoint or mechanism, such as mutations, clastogenic or aneugenic damages. Newly developed assays, such as the BlueScreen™ HC (Hughes et al., 2012), the p53 CALUX^®^ (Van der Linden et al., 2014) or the ToxTracker^®^ (Hendriks et al., 2012) revolve around pathways that are part of the mammalian DNA damage response. These targets are supposed to ensure a response connected to the presence of genotoxic substances and stresses (Feng et al., 2005).

Some important genes and proteins involved in the genotoxicity response of mammalian cells, such as p53, GADD45α, p21 or γH2AX (Watters et al., 2009; Salvador, Brown-Clay & Fornace, 2013) have been the center of studies in previous years. Especially the tumor suppressor protein p53, which is known to be a major checkpoint in the genotoxicity response for mammalian cells, is of great interest (Feng et al., 2005). Further, it is a key regulator of cell senescence, cell survival and cell death, giving important insight in the DNA damage response mechanisms in mammalian cells. This makes it a prime candidate for toxicologically sensitive genotoxicity testing.

Most genotoxicity assays have been used to screen pure chemicals for their toxicological effect (Bopp et al., 2015). However, in recent years the assessment of complex mixtures, such as environmental samples, food contact material (FCM) or plant extracts, instead of pure substances has been of interest and the use of *in vitro* assays for this was recommended by several regulatory bodies (EFSA, 2009; Schilter et al., 2019). In mixtures, there are several compounds present at low concentrations. Therefore, the aim of current *in vitro* assays must also include the detection of substances at low levels. For this, the lowest effective concentration (LEC) value has to be taken into account, which is the lowest concentration of a genotoxin, where a positive result is obtained in a given *in vitro* bioassay. Recent publications cover the subject of analytical sensitivity of some genotoxicity assays (Rainer et al., 2018; Schilter et al., 2019; Pinter et al., 2020) and came to the conclusion that current methods are not sufficient for the analysis of complex mixtures. In this context, analytical sensitivity refers to an assay’s ability to detect substances at low concentrations, meaning low LEC values respond to a high analytical sensitivity and will be referred to as such from now on.

In this study, the aim was to develop a reliable eukaryotic genotoxicity assay for the analysis of complex mixtures. For this purpose, it had to detect a broad range of genotoxic substances correctly, with a high toxicological sensitivity and specificity. Particular emphasis was given on the detection at low concentration levels (=corresponding to low LEC values), as the analytical sensitivity is of great importance for complex mixtures. In order to omit animal derived products, such as S9 liver extract, in this assay, the human liver cell line HepG2 was chosen, as it is p53 competent, has some endogenous metabolizing activity and is highly resistant towards toxic substances (Westerink & Schoonen, 2007b).

## Materials & Methods

In this study 16 known genotoxic substances, 11 non-genotoxic substances and 7 substances with known conflicting results for genotoxicity were tested derived partly from the ECVAM (European Centre for the Validation of Alternative Methods) list (Kirkland et al., 2016).

Known-genotoxic substances (CAS-Nr.; abbreviation): 2-acetylaminofluorene (53-96-3; 2-AAF), actinomycin D (50-76-0), aflatoxin B1 (1162-65-8), benzo-α-pyrene (50-32-8; B αP), cisplatin (15663-27-1), colchicine (64-86-8), cyclophosphamide (6055-19-2), 2,4-diaminotoluene (95-80-7; 2,4-DAT), 7,12-dimethylbenzanthracene (57-97-6; DMBA), doxorubicin (23214-92-8), N-ethyl-nitrosourea (759-73-9; ENU), etoposide (33419-45-0), methyl methanosulfonate (66-27-3; MMS), mitomycin C (50-07-7; MMC), 4-nitroquinoline-n-oxide (56-57-5; 4NQO), sodium arsenite (7784-46-5; SA).

Non-genotoxic substances: amitrole (61-82-5), ampicillin trihydrate (7177-48-2), 2-(chloroethyl)trimethyl-ammonium chloride (999-81-5), diethanolamine (111-42-2), hexachloroethane (67-72-1), d-mannitol (69-65-8), melamine (108-78-1), methyl carbamate (598-55-0), phenformin HCl (834-28-6), pyridine (110-86-1), tris(2-ethylhexyl)phosphate (78-42-2).

*In vitro* false positive non-genotoxic substances: benzyl alcohol (100-51-6), eugenol (97-53-0), 2-ethyl-1,3-hexanediol (94-96-2), D,L-menthol (15356-70-4), sodium saccharin (128-44-9), sulfisoxazole (127-69-5), tert-butylhydroquinone (1948-33-0; tBHQ), urea (57-13-6).

Dulbecco’s modified eagle’s medium (DMEM) and fetal bovine serum (FBS) were purchased through PAN Biotech (Aidenbach, GER), Hyclone™ Pen/Strep 100x solution through GE Healthcare Life Sciences (Buckinghamshire, UK). Pure substances were purchased by Sigma Aldrich (Missouri, USA) and dissolved in dimethyl sulfoxide (DMSO) (Sigma, USA), or in another solvent as indicated. Cisplatin, 2,4-DAT, etoposide, eugenol, d-mannitol, D,L-menthol, phenformin HCl, fluometuron, phenanthrene and progesterone were obtained from Santa Cruz Biotechnology (CA, USA).

### Cell line

HepG2 (ATCC HB-8065, CVCL_0027) cells were stably transfected with a p53 reporter construct using the PiggyBac transposon system (Wilson, Coates & George, 2007). For this, a pGVL8 backbone was used (Mertl et al., 2019), with a six times multimerized p53 binding site from GADD45 (sense: GAACATGTCTAAGCATGCTG) (Hollander et al., 1993). The development of the HepGentox cell line was based on previous reporter optimizations for different signaling pathways (Mertl et al., 2019; manuscript in preparation: Steurer, 2020). A six times multimerized p53 binding site was introduced upstream of an Nluc reporter gene. We chose the short-lived NlucPAU (NanoLuc containing mRNA and protein destabilizing sequences Steurer et al., 2018) to reduce the background signal (= no accumulation) and obtain high induction rates after a short incubation time at lower cytotoxic side effects. The construct was stably integrated into HepG2 cells and one clone was selected as the HepGentox cell line.

The cells were cultivated in DMEM, substituted with 10% FBS and 1% Pen/Strep at 37 °C and 5% CO_2_. Individual clones were raised and tested for their performance. Through initial experiments with a luciferase assay the maximum induction of several clones were tested with a selection of genotoxic substances. In a next step, promising clones were screened concerning their LEC values and the most suitable clone was selected. Cells were frozen at a passage of three and used up to a maximum of 12 passages. The clones were selected by adding puromycin to ensure the stability of the cell line and the inserted construct. Further, induction levels, response of the negative and positive controls and background results were closely monitored throughout the course of this study to test for the cell line’s stability. For testing of pure substances, the cells were seeded at a concentration of 2 × 10^4^ cells/well in a 96 well plate with 100 µL of cell suspension per well. After 24 h, the cells were treated with the genotoxic substance and the following day the cell response was measured. For substance treatment, DMSO was used as a solvent vehicle and applied at a maximum of 1% in DMEM, supplemented with 5% FBS. A maximum substance concentration of 1 mM in the well was chosen. If cytotoxic effects or precipitation/insolubility was observed, the concentrations were altered accordingly.

### Optimisation experiments

For optimisation experiments, the cells were treated with the pure substances 4NQO at a top concentration of 0.63 µM and BαP at 10 µM solved in DMSO. As a vehicle control 1% DMSO was used and the DMSO concentration was steady over the whole plate. To determine the optimal cell concentration, the cells were cultivated as described above and 100 µL of a cell suspension was seeded in 96 wells plate with 1 × 10^4^, 2 × 10^4^, 4 × 10^4^, 6 × 10^4^, 8 × 10^4^ and 1 × 10^5^ cells/well. The cells were treated with 4NQO and BαP and incubated for 24 h before measurement. For incubation time experiments, the cells were seeded at 2 × 10^4^ cells/well in a 96 well plate and incubated with 4NQO and BαP for 2, 6, 24, 48 or 72 h until measurement. To determine the optimal FBS concentration, the cells were seeded at 2 × 10^4^ cells/well in a 96 well plate and treated with 4NQO or BαP solved in DMEM supplemented with 5%, 10% and 15% FBS for each plate and measured after 24 h of incubation. For DMSO experiments, 2 × 10^4^ cells/well were seeded in a 96 well plate and treated with 4NQO and BαP solved in DMEM. Over half a plate, a DMSO concentration of 0.25, 0.5, 1.0, 1.5 and 2.0% was ensured and the vehicle control was adjusted accordingly. Measurements were done after 24 h of incubation.

### Measurement

Viability was determined using a resazurin assay as described previously (Riegel et al., 2017) prior to luciferase measurement with a multiplate reader Infinite^^®^^200 Pro (Tecan, CH). single NanoLuc measurement was performed as described in *Steurer* et al. (2018) using a Luminoskan™ Microplate Luminometer (Thermo Fisher, Waltham, MA, USA). For viability measurement, resazurin was diluted in 1xPBS and added in the wells to a final concentration of 5 µM in the plate. The plates were further incubated with the resazurin for 1 h, before measurement with an Infinite^^®^^200 Pro (Tecan, CH) multiplate reader at excitation wavelength 540 nm and emission wavelength 590 nm. For viability, a threshold of 70% was used. For evaluation, a threshold of 1.7 was applied, which was determined through statistical analysis of blank values (= vehicle control with 1% DMSO) by addition of three times the standard deviation. In these experiments a fold induction of 0.7 for the vehicle control was found with a standard deviation of 0.312. This data was obtained from two individual blank experiments (192 wells in total) and the background data of 113 experiments (12 wells each). A fold induction of a substance or sample above the threshold of 1.7 was considered as positive.

### S9 experiments

For metabolization experiments, 1254 aroclor induced S9 rat liver extract was used (Moltox, NC, USA) and cofactors nicotinamide adenine dinucleotide phosphate (NADPH), Glucose-6-Phosphate (G6P) and MgCl_2_ were purchased from Carl Roth (Karlsruhe, GER) and Glucose-6-Phosphate-Dehydrogenase (G6P-DH) from Sigma Aldrich (US). Two different S9 protocols were followed, with different S9 composition depending on the incubation time with the S9 mixture. Final concentration of the compounds in the wells were: 5 mM MgCl_2_, 3 mM G6P, 0.2 mM NADPH, 0.3 units/mL G6P-DH and 330 µg/mL (3 h protocol) or 10 µg/mL (24 h protocol according to Mollergues et al. (2016)) of S9 liver extract. Cells were either treated for 3 h with a higher concentrated S9 mix, then washed with Dulbecco’s phosphate buffered saline (DPBS) and further incubated with DMEM containing 5% FBS and 1% DMSO for another 21 h. Alternatively, treatment was done for 24 h with a lower concentrated mix, without a change of medium. Luciferase and resazurin measurements were conducted the same way as without S9 addition. The 1254 aroclor induced S9 rat liver extract was used simultaneously in the same laboratory for the Ames MPF™ assay to prove its functionality.

### Complex mixtures

For testing of complex mixtures, the cells were cultivated as described above and treated with 1% of an FCM sample migrate solved in DMSO. The FCM migrate was produced through migration and concentration of polyethylene, following the protocol by Rainer et al. (2019). Upon addition to the HepGentox, the sample was spiked with 4NQO or BαP in a range where a positive response was expected. The spikes were solved in DMEM with additional 1% DMSO, therefore the DMSO concentration remained at 1% over the whole plate.

## Results –Assay Optimization

The goal of this study was to develop a eukaryotic assay with improved LEC values to detect pure substances at the lowest concentration possible in complex mixtures. Apart from optimizing the reporter construct, the assay conditions should be adapted for this purpose. For finding the optimal assay conditions, two representative genotoxic substances were chosen namely 4NQO and BαP. Both 4NQO and BαP are directly acting genotoxins, but while 4NQO does not need any metabolization, BαP unfolds its genotoxic potential only upon the presence of an exogenous metabolizing system. With these two substances the influence of the assay parameters: cell number, incubation time, FBS and DMSO concentration as well as the protocol for external metabolic activation (S9 treatment) were analyzed in the following subchapters.

### Results – assay optimization –cell number and incubation effects

**Figure 1:**
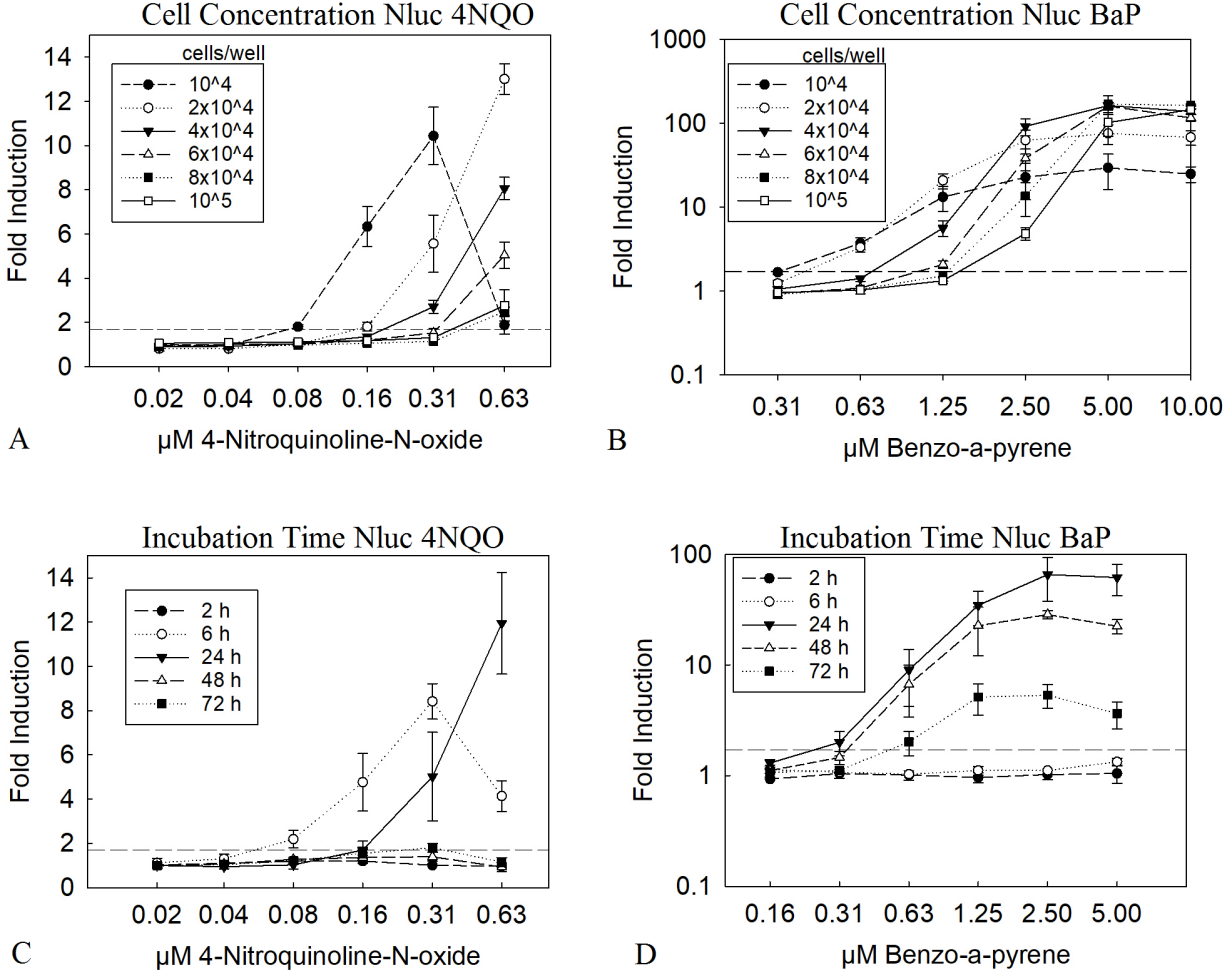
Optimization of cell number and incubation time. The diagrams A and B show the Nluc measurement of experiments with different cell concentrations treated with 4NQO (A) and BαP (B) for 24 h. Diagrams C and D show 2 × 10^4^ cells/well treated with 4NQO (C) and BαP (D) for 2, 6, 24, 48 and 72 h. *X*-axis represents the concentration of the genotoxic substances and *y*-axis the fold induction of the sample, which was calculated with the mean sample value divided by the mean background (1% DMSO). The dashed line indicates the threshold of 1.7 (background + 3 times standard deviation), above which the first signal was taken as LEC value. Experiments were conducted in triplicates, error bars represent standard deviation. The data show the mean of at least three independent experiments with twelve replicates each.

A low cell number is leading to a higher amount of substance per cell. To observe if this can be directly translated into a lower LEC value in the assay we tested 10,000 to 100,000 cells per well in a 96 well plate. The results in Figs. 1A and 1B clearly show, that a low cell number led to a LEC value of 0.16 µM for 4NQO and 0.63 µM for BαP, compared to the highest cell concentration of 100,000 cells per well, with four times higher LEC values of 0.63 µM and 2.5 µM, respectively. This was the case for both substances; which may or may not need metabolic activation. Of course, a higher amount of substance per cell might also result in greater cytotoxicity, therefore viability was closely observed in parallel. A threshold of 70% was taken as a limit for the viability. For 4NQO, this limit was reached earlier with lower cell concentrations (2 to 4-fold compared to higher cell concentrations). However, for B*α*P, the viability was stable through all concentrations (Figs. S1A and S1B). A concentration of 2 × 10^4^ cells/well was chosen as optimum, as here the LEC value was low at 0.31 µM for 4NQO and 0.63 µM for BαP. Further, the viability was considered to be reasonably stable at higher concentrations of genotoxic substances as it remained above the 70% threshold.

Genotoxic substances have very heterogeneous chemical properties and therefore cover a wide variety of modes of action (MoA). Further, the MoA together with differences in the kinetics of the cellular uptake greatly influences the kinetics of the induced DNA damage and the cellular response. To analyze the influence of the incubation time on the resulting LEC values, the HepGentox cells were tested after 2, 6, 24, 48 and 72 h treatment with the model substances 4NQO or BαP (Figs. 1C and 1D). The experiment clearly showed that substances, which have a genotoxic effect independent of a metabolic activation system, such as 4NQO affected the cells shortly after substance treatment, as a signal could already be seen after 6 h with a LEC value of 0.08 µM and after 24 h with a LEC of 0.16 µM (Fig. 1C). However, at later time points induction above the threshold was no longer observed. Contrary, for BαP a signal was observed only after 24 h with a LEC of 0.31 µM or more (Fig. 1D). Further, viability dropped at higher 4NQO concentrations with increasing time at 0.31 µM below the 70% threshold, which was also observed for BαP at a concentration of 0.63 µM after 48 and 72 h of incubation (Fig. S1). This leads to the conclusion that an incubation time of 24 h is the most reasonable, since only at this measurement point both substances, which act genotoxic with and without a metabolic activation system, could be detected.

### Results –assay optimization –serum and DMSO effects

**Figure 2:**
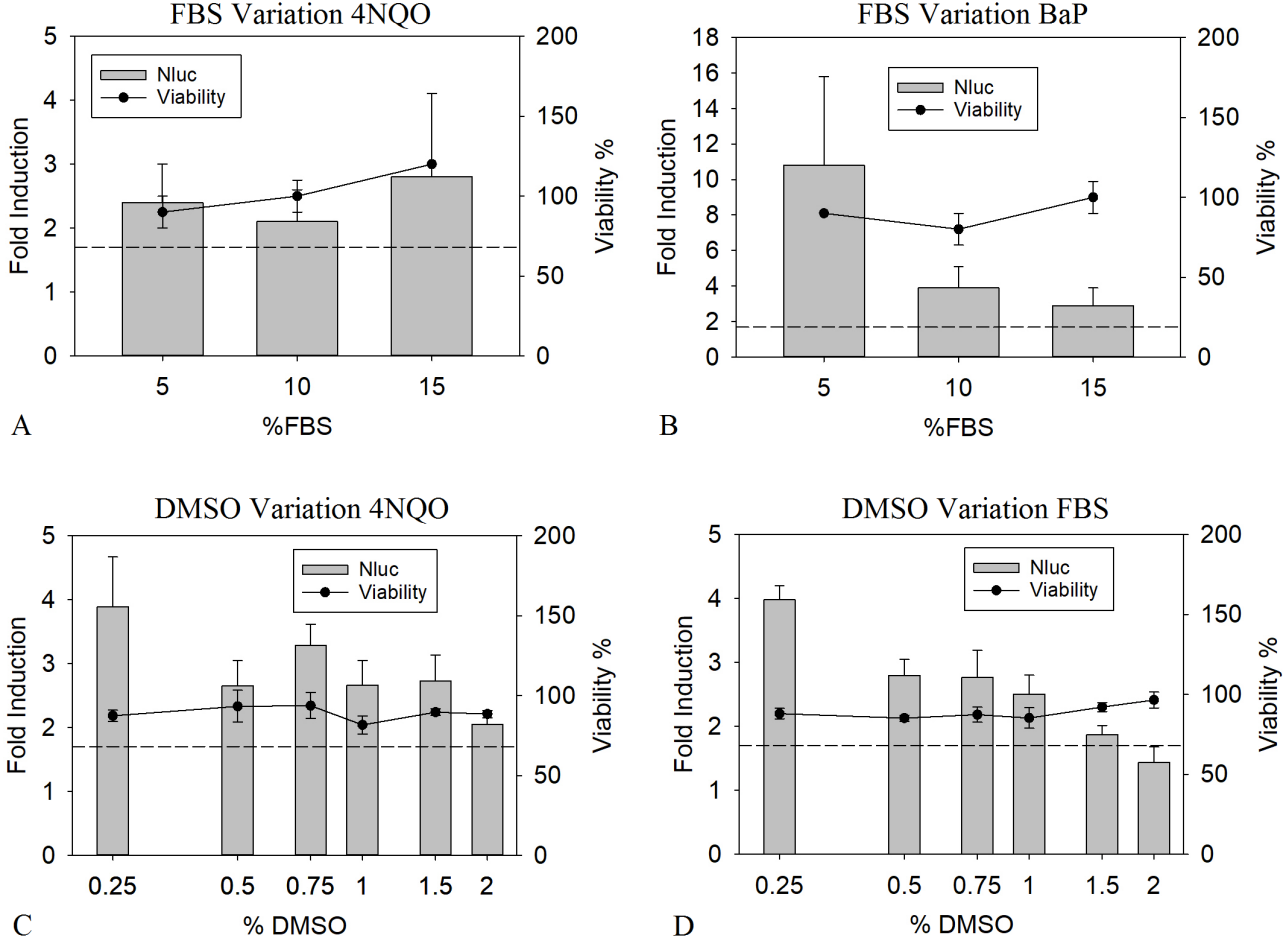
Optimization of FBS and DMSO concentrations. Nluc measurement after 24 h of cells treated with the pure substances 0.16 µM 4NQO (A and C) and BαP (0.63 µM in B and 0.31 µM in D) with 5, 10 or 15% FBS (A and B) or in the presence of 0.25, 0.50, 0.75, 1.00, 1.50 or 2.00% DMSO (C and D). *Y*-axis in A and C show the fold induction, calculated by dividing the Nluc value by the mean background (1% DMSO). *Y*-axis in B and D show the viability compared to the background (= 100% viability). Experiments were conducted in triplicates. The dashed line indicates the threshold of 1.7 (background + 3 times standard deviation). The data show the mean of at least three independent experiments with twelve replicates each.

Supplementation of cell culture media with serum is known to greatly benefit the cell viability (OECD, 2018). However, binding of genotoxic substances to serum proteins might negatively influence the LEC values, as this leads to a reduction of free available compounds (Craig & Kunin, 1976). To analyze the influence of the presence of serum proteins on the toxicological and analytical sensitivity of the HepGentox assay, 0.16 µM of 4NQO and 0.63 µM of BαP were tested in the presence of different serum concentrations, namely 5, 10 and 15% FBS. Preliminary experiments (data not shown) found these concentrations to be suitable, since lower amounts of FBS led to a decrease in viability or a reduction in proliferation in the control culture. Therefore 5% FBS was used as a minimum level. As shown in Figs. 2A and 2B between the various FBS concentrations, no apparent differences could be found for the LEC values with 4NQO. However, the induction of 5% FBS was elevated by a factor of 2.5 to 3.5 for BαP compared to the other concentrations and was therefore chosen as optimum.

When analyzing the genotoxicity of complex mixtures, the application of a maximum amount of sample is of interest to increase the substance concentration in the assay. Unfortunately, most samples of complex mixtures are not aqueous, but solved in organic solvents not tolerated well by mammalian cell culture cells such as DMSO. For mammalian cells, the DMSO compatibility usually ranges around 0.5 to 2%, greatly limiting the sample application (Timm et al., 2013). To determine the DMSO tolerance in the HepGentox assay the cells were treated either with 0.16 µM 4NQO or 0.31 µM BαP dissolved in 0.25, 0.50, 0.75, 1.00, 1.50 or 2.00% DMSO. Figs. 2C and 2D show that upon increasing concentration of DMSO with 4NQO a quenching of the signal was observed by 50% from the highest induction at 0.25% DMSO to the lowest signal at 2% DMSO, therefore possibly leading to higher LEC values. The same was observed with BαP, where the signal was reduced by 75% from its highest peak at 0.25% DMSO to its lowest at 2% DMSO. Contrary, the viability was not reduced at any tested concentration. At a DMSO concentration of 0.25% the highest induction levels could be observed. Nevertheless in regards of the research question, this concentration is not ideal for sample testing. Due to the fact, that this leads to a higher sample dilution and therefore indirectly increasing the LEC values when a sample is added. In terms of correlating sample input, viability and quenching effect, 1% DMSO was chosen as assay condition. This is a holistic approach so that the results of the determined LEC values can be directly compared to the sample testing.

### Results –assay optimization –external metabolizing system

Many genotoxic substances need metabolic activation, which is normally achieved via the application of S9 rat liver extract in *in vitro* assays. The use of S9 does not only raise ethical questions, but is also expensive and due to cytotoxicity and variation of substrate quality its use is discussed (Jacobs et al., 2013). Further, more sample volume and laboratory time is necessary, as testing has to be done with and without the addition of S9, since it possesses both activating and detoxifying abilities, which could lead to false negative results. In this study, two different S9 protocols (incubation for 3 h with 330 µg/mL and 24 h with 10 µg/mL S9) as proposed by Mollergues et al. (2016) were tested, as well as the ability of the HepGentox cell line to metabolize the substances without S9 addition. Results were evaluated for LEC values, as well as for viability (Table 1 and and Figs. S2 and S3). The results showed that HepGentox cells tolerate both S9 treatments well, as the viability was hardly compromised (Fig. S3).

**Table 1:** Results of the HepGentox assay with different S9 protocols. HepGentox cells were incubated without S9, for 3 h with 330 µg/mL S9 or for 24 h with 10 µg/mL. LEC results for the respective protocols are given and the viability at the LEC value or for the highest applied concentration when no positive result could be obtained for this substance with the protocol.

Requires Metabolization (Kirkland et al., 2016)	Substance	S9 Protocol	LEC [µM]	Viability for LEC value or highest concentration
No	Cisplatin	24 h with no S9 mix added	1.25	90%
		3 h with 330 µg/mL S9 mix	Negative	70%
		24 h with 10 µg/mL S9 mix	Negative	90%
No	N-Ethyl-nitrosourea	24 h with no S9 mix added	625	90%
		3 h with 330 µg/mL S9 mix	625	110%
		24 h with 10 µg/mL S9 mix	Negative	100%
Yes	2-Acetylaminofluorene	24 h with no S9 mix added	Negative	80%
		3 h with 330 µg/mL S9 mix	Negative	70%
		24 h with 10 µg/mL S9 mix	Negative	60%
Yes	Aflatoxin B1	24 h with no S9 mix added	0.63	90%
		3 h with 330 µg/mL S9 mix	0.31	60%
		24 h with 10 µg/mL S9 mix	Negative	70%
Yes	Benzo- α-pyrene	24 h with no S9 mix added	0.63	100%
		3 h with 330 µg/mL S9 mix	1.25	60%
		24 h with 10 µg/mL S9 mix	Negative	80%
Yes	Cyclophosphamide	24 h with no S9 mix added	Negative	50%
		3 h with 330 µg/mL S9 mix	625	90%
		24 h with 10 µg/mL S9 mix	Negative	70%
Yes	2,4-Diaminotoluene	24 h with no S9 mix added	2,500	100%
		3 h with 330 µg/mL S9 mix	Negative	30%
		24 h with 10 µg/mL S9 mix	Negative	30%
Yes	Etoposide	24 h with no S9 mix added	2.5	60%
		3 h with 330 µg/mL S9 mix	Negative	100%
		24 h with 10 µg/mL S9 mix	Negative	60%

Concerning the LEC values, the 3 h protocol was more promising than the 24 h protocol without S9, since the LEC values were improved for aflatoxin B1 by a factor of two. For cyclophosphamide, (negative after 24 h to 625 µM with the 3 h protocol) the viability was hardly affected. However, for other substances there were no improvements or positive signals. It can be seen that substances needing a metabolizing system, show a response within the same order of magnitude (e.g., aflatoxin B1 with a LEC of 0.63 µM without S9 and 0.31 µM after 3 h with S9, ENU with a LEC of 625 µM for both with/without S9) or better (e.g., BαP with a LEC of 0.63 µM without S9 and 1.25 µM after 3 h with S9) LEC value. Further, the metabolizing activity does not compromise its ability to detect substances that might be negative with S9, such as cisplatin. However, it has to be noted that the substance cyclophosphamide would not have been detected without the addition of S9. Since the assay was developed to detect possible genotoxic substances at low concentration, it was considered as negligible that cyclophosphamide could not be detected without S9, as the LEC value was very high with 625 µM and close to the testing threshold of 1 mM.

### Results –pure substances testing

For pure substances testing, a pool of known genotoxic and non-genotoxic substances was chosen from the updated ECVAM list (Kirkland et al., 2016) and some genotoxins of interest were added as well (e.g., 4NQO, actinomycin C). Overall, 16 known genotoxins, 11 known non-genotoxins and 7 non-genotoxins that tend to give positive results in *in vitro* tests (false positives) were tested. Substances were analyzed up to a top concentration of 1 mM or until the viability dropped below 70%. The maximum concentration of 1 mM was used to prevent the rise of false positive substances, as was proposed by Kirkland et al. (2007). Upon precipitation, insolubility of the stock or cytotoxic effects, a lower concentration was chosen. A threshold of 1.7 fold induction compared to the blank was used, which was calculated from a broad series of negative controls adding three times the standard deviation. For negative substances, a positive control of 2 µM 4NQO and a vehicle control of 1% DMSO was used. The maximum fold induction over the concentration range is given in Tables 2 and 3 as maximum IF. This is the ratio of the mean Nluc response compared to the background signal. The assay proved to have sufficient maximum inductions compared to the background, proving that a genotoxic response leads to a consistent increase in signal intensity in the HepGentox making the assay robust in its response. Overall, a toxicological sensitivity of 87.5% (14 out of 16) and a specificity of 94% (17 out of 18) was achieved as can be seen in Tables 2 and 3. This is within the range of current reporter gene assays dealing with genotoxicity, such as the BlueScreen™ HC with 80% sensitivity and 100% specificity (Hughes et al., 2012) and the p53 CALUX^®^ with 82% and 90% (Van der Linden et al., 2014).

**Table 2:** Results of the 16 tested known genotoxins to cause *in vitro* positive results with the HepGentox. The sample solvent is indicated and the first positive result above the threshold of 1.7 was taken as LEC value. A negative result means no induction above the threshold was observed. The maximum fold induction (IF) over the concentration range is given, not taking cytotoxicity into account.

	**Substance**	**CAS**	**Solvent**	**LEC [µM]**	**LEC [µg/mL]**	**Max IF**
Known *in vitro* and *in vivo* genotoxic substance	Cyclophosphamide	6055-19-2	DMSO	313	88 (+S9)	38.64
	N-Ethyl-nitrosourea	759-73-9	DMSO	625	73	17.94
	Methyl methanosulfonate	66-27-3	H2O	625	69	1.95
	Benzo-a-pyrene	50-32-8	DMSO	0.6	0.2	75.57
	7,12-Dimethylbenzanthracene	57-97-6	DMSO	1.6	0.4	3.19
	2-Acetylaminofluorene	53-96-3	DMSO	Negative	Negative	1.07
	2,4-Diaminotoluene	95-80-7	DMSO	625	76	10.39
	Aflatoxin B1	1162-65-8	DMSO	0.6	0.2	17.02
	Cisplatin	15663-27-1	DMSO	0.6	0.2	19.39
	Sodium arsenite	7784-46-5	H2O	100	13	5.82
	Etoposide	33419-45-0	DMSO	1.3	0.8	4.01
	4-Nitroquinoline-n-oxide	56-57-5	DMSO	0.2	0.04	10.49
	Colchicine	64-86-8	DMSO	Negative	Negative	1.65
	Mitomycin C	50-07-7	DMSO	0.4	0.1	9.53
	Actinomycin D	50-76-0	DMSO	1.3	1.6	14.03
	Doxorubicin	23214-92-8	DMSO	0.06	0.03	279.32

**Table 3:** Results of the 11 known non-genotoxins and 7 non-genotoxins known to cause *in vitro* positive results. The sample solvent is indicated and the first positive result above the threshold of 1.7 was taken as LEC value. A negative result means no induction above the threshold was observed. The maximum fold induction (IF) over the concentration range is given, not taking cytotoxicity into account.

	**Substance**	**CAS**	**Solvent**	**LEC**	**Max IF**
Known non-genotoxic substances	Ampicillin trihydrate	7177-48-2	H2O	Negative	1.10
	d-Mannitol	69-65-8	DMSO	Negative	1.16
	Phenformin HCl	834-28-6	DMSO	Negative	1.11
	(2-Chloroethyl)trimethyl- ammonium chloride	999-81-5	DMSO	Negative	1.03
	Amitrole	61-82-5	DMSO	Negative	1.15
	Diethanolamine	111-42-2	DMSO	Negative	1.23
	Melamine	108-78-1	DMSO	Negative	1.06
	Methyl carbamate	598-55-0	DMSO	Negative	1.03
	Pyridine	110-86-1	DMSO	Negative	1.03
	Tris(2-ethylhexyl)phosphate	78-42-2	96% Ethanol	Negative	1.03
	Hexachloroethane	67-72-1	DMSO	Negative	1.19
*In vivo* negatives, sometime *in vitro* positives	D,L-Menthol	15356-70-4	DMSO	Negative	1.08
	2-Ethyl-1,3-Hexanediol	94-96-2	DMSO	Negative	1.13
	Sulfisoxazole	127-69-5	DMSO	Negative	1.66
	Urea	57-13-6	DMSO	Negative	1.22
	Sodium Saccharin	128-44-9	DMSO	Negative	1.26
	Eugenol	97-53-0	DMSO	Negative	1.18
	Tert-butylhydroquinone	1948-33-0	DMSO	10 µg/mL 63 µM	4.08

**Table 4:** Comparison of the HepGentox assay to regulated and OECD approved (OECD, 2014b, OECD, 2014a) mammalian genotoxicity assays.

**Substance**	**CAS**	**HepGentox [µg/mL]**	**Micronucleus [µg/mL]**		**Comet [µg/mL]**		**Ames [µg/mL]**
Cyclophosphamide	6055-19-2	88	9 (-)^1^	HepG2	70 (+)^4^	Human blood cells	0.74 (+)^15^
N-Ethyl-nitrosourea	759-73-9	73	73 (-)^2^	HepaRG	250 (-)^5^	TK6	12 (-)^16^
Methyl methanosulfonate	66-27-3	69	11 (-)^1^	HepG2	8 (-)^6^	Human blood cells	0.5 (-)^17^
Benzo-a-pyrene	50-32-8	0.2	3 (-)^1^	HepG2	1.3 (+)^7^	MRC5CV1	0.21 (+)^17^
7,12-Dimethylbenzanthracene	57-97-6	0.4	2 (-)^2^	HepaRG	0.3 (+)^7^	MRC5CV1	7.8 (+)^18^
2-Acetylaminofluorene	53-96-3	Negative	58 (-)^2^	HepaRG	Negative (-)^8^	HepG2	0.1 (+)^17^
2,4-Diaminotoluene	95-80-7	76	39 (-) ^1^	HepG2	178 (-)^9^	HepG2	0.02 (+)^19^
Aflatoxin B1	1162-65-8	0.2	0.08 (-)^2^	HepaRG	9.4 (+)^10^	HepG2	0.001 (+)^17^
Cisplatin	15663-27-1	0.2	95 (-)^1^	HepG2	Negative (-)^6^	Human blood cells	0.37 (-)^16^
Sodium arsenite	7784-46-5	8	0.1 (-)^1^	HepG2	26^*^^11^	Human blood cells	N/A
Etoposide	33419-45-0	0.8	2 (-)^1^	HepG2	10 (-)^12^	Human blood cells	185 (-)^20^
4-Nitroquinoline-n-oxide	56-57-5	0.03	0.6 (-)^2^	HepaRG	0.01 (-)^7^	MRC5CV1	0.004 (-)^21^
Colchicine	64-86-8	Negative	5 (-)^3^	AHH-1, MLC-5	N/A		N/A
Mitomycin C	50-07-7	0.1	N/A		Negative (-)^13^	TK6	N/A
Actinomycin D	50-76-0	1.6	N/A		N/A		N/A
Doxorubicin	23214-92-8	0.03	0.05 (-)^1^	HepG2	0.05 (-)^14^	Human blood cells	N/A

**Notes.**

(+) value obtained with S9 addition.

(-) value obtained without S9.

* no information given whether an exogenous metabolizing system was used to obtain the result.

N/A no LEC data was found in the literature for a substance with the respective assay.

1 Westerink et al. (2011).

2 Le Hégarat et al. (2014).

3 Parry et al. (1996).

4 Hartmann et al. (1995).

5 Kawaguchi et al. (2010).

6 Pfuhler & Wolf (1996).

7 Speit & Hartmann (1995).

8 Valentin-Severin et al. (2003).

9 Séverin et al. (2005).

10 Corcuera et al. (2011).

11 Hartmann & Speit (1996).

12 Lebailly et al. (1997).

13 Henderson et al. (1998).

14 Anderson, Yu & Browne (1997).

15 Eliopoulos, Mourelatos & Dozi-Vassiliades (1995).

16 Zeiger et al. (1992).

17 Kenyon et al. (2007).

18 Kaden, Hites & Hilly (1979).

19 Ames, McCann & Yamasaki (1975).

20 Nakanomyo, Hirokam & Shiraya (1986).

21 Blahová, Lahitová & Sokolík (1997).

In a next step, the newly developed HepGentox assay was compared to the LEC values found for other commonly used mammalian assays, for genotoxicity testing, as can be seen in Table 4. The micronucleus has been recommended as part of a test battery for genotoxicity testing in regulatory guidelines (EFSA, 2011; ICH, 2012) and has been approved and standardized by the OECD (OECD, 2014b; OECD, 2014a). The comet test is included here as well, which is also an *in vitro* assay used for the detection of DNA breaks and damages, especially for clastogenic substances (Pfuhler & Wolf, 1996). For the comet assay, an OECD guideline exists only for the *in vivo* method (OECD, 2014b), but it can also be used for *in vitro* testing for genotoxicity. Since for the micronucleus and the comet different cell lines can be used not all substance LEC values could be found for HepG2 cells. Therefore, the used cell line for the LEC result is given in Table 4. Finally, the Ames test is also shown in Table 4, which is an assay used for the detection of direct DNA-reactive substances and especially for mutagens. The Ames test is widely applied and recommended by regulatory guidelines and standardized by the OECD (OECD, 1997). The results obtained for the HepGentox were based on the results in Table 2 and the LEC values for the micronucleus, the comet assay and the Ames test were taken from a literature survey. Comparing the results to several assays is challenging, as there is limited data for several substances and assays. We decided to compare the assays in groups and only for the data where a literature result was available for the assay group. Out of the 15 substances in Table 4, the HepGentox proved to have lower LEC values for 26% (4 out of 15) when looking at the micronucleus and the comet assay. Specifically, for cisplatin the HepGentox was 500 times more sensitive than the comet or the micronucleus tests. For 20% of the substances, higher LEC values were observed with the HepGentox by a factor of two to ten and for 54% the assay was within the range of the others. When comparing the HepGentox to the Ames test in Table 4 it can be seen that the mammalian assay only led to lower LEC values for the substances 7,12-DMBA and etoposide. For the other substances, the Ames test had superior LEC values, which was already observed in a literature survey by Pinter et al. (2020). When looking at the reporter gene assays in Table S1, the BlueScreen™ HC and the p53 CALUX^®^, we found that the HepGentox had lower LEC values for 38% (5 out of 13) of the substances. For other substances, it performed in an equal concentration range detecting 31% (4 out of 13) with a similar LEC when compared to both assays, but 31% had a higher LEC than the BlueScreen^TM^ HC or the p53 CALUX^®^. To sum up it can be seen that by comparing the HepGentox to the other genotoxicity assays, it can be found that all of these assays have their advantages and disadvantages when it comes to the analytical sensitivity of the assay, namely the LEC value. However, the HepGentox is the only assay, which has been specifically designed and evaluated for the application of complex mixtures. This makes it an interesting assay, compared to previous test systems exclusively designed for pure substances testing and could be incorporated into a comprehensive test battery together with chemical analysis and other *in vitro* bioassays, such as the Ames test.

### Results –assay application –complex mixtures and cytotoxicity

**Figure 3:**
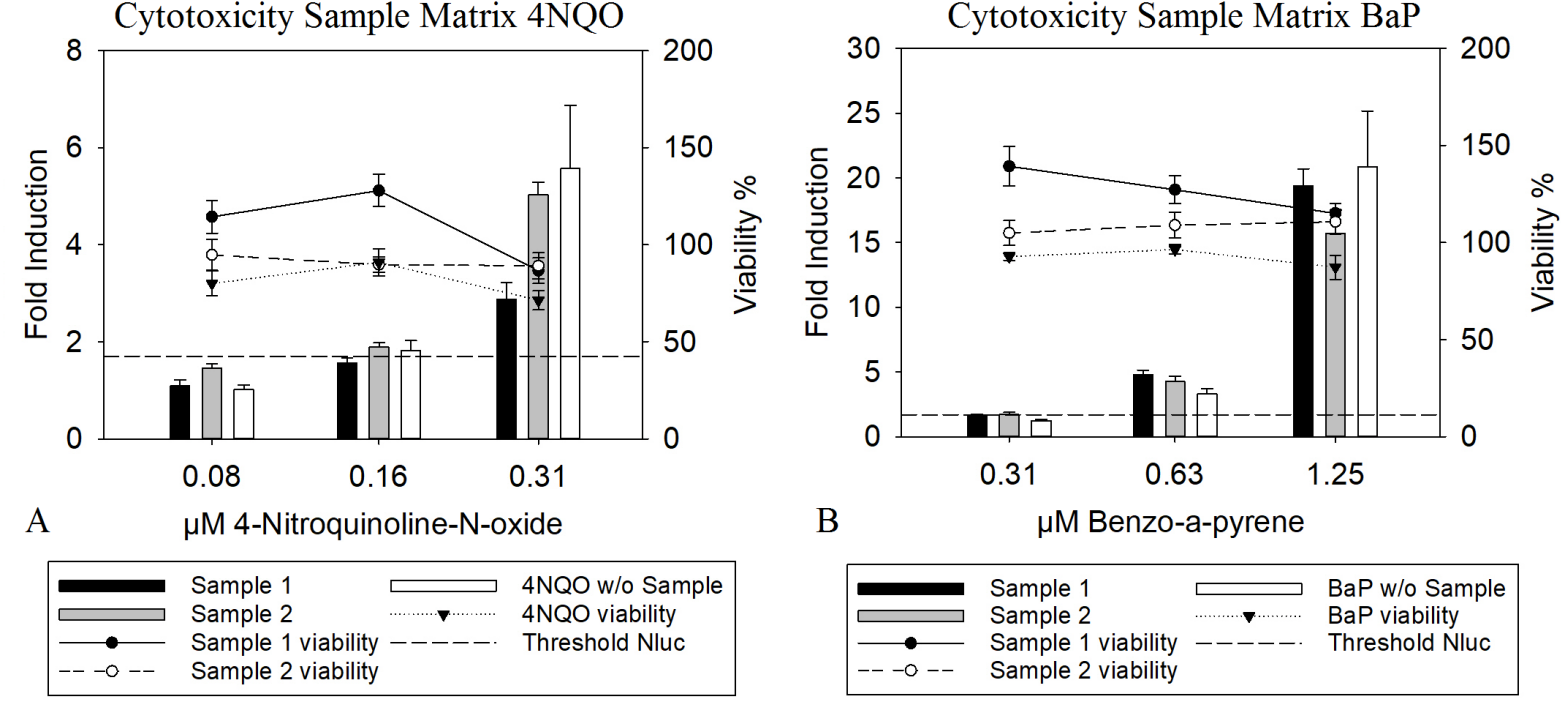
Nluc and resazurin measurement of cells treated with complex mixtures. The cells were treated with 1% sample with DMSO as a solvent and with 4NQO (A) or BαP (B) as positive substances. The Nluc induction was calculated as the mean luciferase activity divided by the background value resulting in a fold induction, indicated for the different concentrations of the genotoxic substance. The threshold of 1.7 (background + 3 times standard deviation, shown as dashed line) was used to determine the LEC, which is the first concentration above it. For viability measurement, the metabolisation of resazurin compared to the blank value was used. Here the threshold was 70% indicating that values above had a higher viability. The data show the mean of at least three independent experiments with twelve replicates each.

The presence of a complex mixture matrix was an important aspect during development and validation of the assay, since the test application should include the analysis of complex mixtures. For these mixtures, so called matrix effects are crucial as they can strongly affect the outcome of a result and its reliability (Schilter et al., 2019). To determine the ability of the assay to detect genotoxic substances in the presence of a complex matrix, spiking experiments were conducted. For this, FCM polyethylene extracts solved in DMSO were used, to simulate the presence of a complex matrix and were spiked with 4NQO and BαP in different concentrations. Further, the viability was regarded more closely, since a cytotoxic effect of substances present in the matrix or mixture might mask a genotoxic effect. The results in Fig. 3A and 3B show that the presence of a complex mixture matrix did have an effect, as the induction for each sample differed slightly. However, the LEC was not affected for BαP and slight alterations were found for 4NQO, as the LEC varied by a factor of two, which is considered to be within the range of biological variation within the assay. Further. when compared to the signals observed for the pure substance without samples, no remarkable deviation could be observed. Moreover, the matrix did not interfere negatively with the cell’s viability. This leads to the conclusion that the presence of a complex mixture matrix is not likely to have any adverse effects regarding the detection of genotoxic substances.

## Discussion

The testing of genotoxicity is an important aspect and ongoing challenge when assessing pure substances and mixtures alike. Unlike carcinogenicity, which has to be tested with long and short term *in vivo* testing to obtain reliable results, as there are several complex mechanisms interacting (Graziano & Jacobson-Kram, 2015), genotoxicity is by now well studied in *in vitro* test systems. Referring to the 3R principals of animal testing (Russell & Burch, 1959) the ECVAM is recommending *in vitro* assays instead of *in vivo* assays and there are already several OECD guidelines for *in vitro* assays to detect genotoxicity of pure substances available (Holley et al., 2017).

In the present study, HepG2 cells were used to establish a reporter-gene assay to detect genotoxic substances reliably in complex mixtures at low concentrations. HepG2 cells have been the focus of numerous genotoxicity studies and a great amount of knowledge has been collected (Valentin-Severin et al., 2003; Steinberg, 2013). A study by Fowler et al. (2012) raised the importance of carefully selecting a mammalian cell line for genotoxicity testing. Ideally, the cell line should be p53 competent (Honma & Hayashi, 2011) and robust towards cytotoxic compounds, so that misleading false positive or negative results can be minimized. HepG2 cells have proven to be somewhat metabolically active, have a functional active p53 protein and produce good results for toxicological sensitivity and specificity (Séverin et al., 2005; Steinberg, 2013 [p50]). As this is an adherent cell line, the HepG2 cells can be used in several genotoxicity assays. For example, the same cells used for the luciferase measurement could also be taken for microscopical micronucleus assessment (OECD, 2014a) providing further important information. In general, HepG2 cells are considered to have a robust viability and are less likely to be affected by cytotoxic effects than other commonly used cell lines (Steinberg, 2013). A drawback of the HepG2 cells was observed by Fowler et al. (2012), as HepG2 cells have a high and variable background when performing the micronucleus and this could lead to the masking of weak positive responses. Moreover, it has been reported that different HepG2 cell lines have a different toxicological sensitivity in the micronucleus (Fowler et al., 2014), which might also be the case for other assays based on this cell line. Further, the conditions of the cell culture are important, since any change in karyotype or viability can greatly affect the cell state concerning metabolisation and consequently the experimental outcome. Nevertheless, promising results have been found with this cell line (Valentin-Severin et al., 2003) and the cell system was considered useful for genotoxicity assessment by the ECVAM (Kirkland et al., 2007), but more research on this is required.

With 87.5% toxicological sensitivity and 94% specificity we consider this assay to be within the range of other mammalian genotoxicity assays, such as the BlueScreen™ HC with 80% and 100% (Hughes et al., 2012) or the p53 CALUX^®^ with 82% and 90% (Van der Linden et al., 2014). The toxicological sensitivity and specificity of the micronucleus tends to vary and is regarded to be prone towards false positive results (Pinter et al., 2020). As Pinter et al. (2020) found, novel reporter gene based assay systems tend to perform very well when it comes to these toxicological parameters. Especially, the specificity of such reporter gene based systems are high, therefore it is unlikely that false positive results might be generated. A study by Kirkland et al. (2005) found that the combination of the Ames test with more than two mammalian assays led to an increase in false positive results. However, study dealt with assays such as the micronucleus, the comet or the MLA. With novel assays, such as reporter gene assays, this is unlikely, as they tend to have a high specificity (Pinter et al., 2020) making a combination possible. The specificity and toxicological sensitivity of the HepGentox can be considered very high, but some false results could be found nevertheless. One false negative substance with the HepGentox was 2-AF, which was also negative in other HepG2 reporter gene assays (Steinberg, 2013 [p.253]), possibly indicating that this substance cannot be detected with this cell line. Further, if a positive result could be obtained then the induced signal was very low and weakly positive at high concentrations. The substance 2-AF is known to induce the AhR pathway, but it is far less active than other amines (Juricek et al., 2014). The other false negative substance with the assay was colchicine, which is known to be aneugenic (Kirkland et al., 2016). Colchicine is known to upregulate the p53 pathway in HepG2 cells, but it has also shown to act independent of p53 in various liver cells (Feng & Kaplowitz, 2000). A longer incubation time could have been necessary to detect aneugenic effects. The false positive tBHQ has been reported to be an issue for HepG2 cell lines, since it was positive for erroneous micronuclei induction in a study by Fowler et al. (2012).

In general, *in vitro* bioassays are commonly used as high throughput screening tools for a variety of applications. In terms of genotoxicity assessment, the use of bioassays is recommended to obtain information and often to determine whether *in vivo* testing is necessary. For medical devices, this is mentioned in the ISO 1993-3:2014 (ISO, 2014), where extracts can be analyzed with eukaryotic or prokaryotic systems for genotoxicity and cytotoxicity. Further, for botanical extracts (EFSA, 2009), novel foods (EFSA, 2016) or FCMs (Schilter et al., 2019) the application of *in vitro* bioassays has been recommended by regulatory bodies and guidelines as well. This is also the case for cosmetic products, where bioassays have been suggested to test for example for dermal absorption, acute toxicity or skin sensitizing effects (SCCS, 2018).

Most genotoxicity assays were specifically developed to perform well in sense of toxicological sensitivity and specificity. This assay, on the other hand, should also consider the analytical sensitivity. With this in mind, the requirements for the HepGentox were to detect known genotoxins and non-genotoxins correctly and at low concentrations. When comparing the LEC values to literature results of other regulatory recommended mammalian genotoxicity assays, such as the micronucleus or the comet assay, 26% of the substances could be detected at lower concentrations and 54% were found in a similar range. These results show that the HepGentox performs well in the area of analytical and toxicological sensitivity and specificity compared to regulatory test systems. However, improvements of the LEC values are still necessary to meet the regulatory recommendations and thresholds proposed (Schilter et al., 2019; Pinter et al., 2020).

Another important factor for the development of the assay is the metabolization of substances through the HepG2 cells itself or with the help of an exogenous system. Since the use of S9 is controversial, it should be limited in *in vitro* assays. Initiatives have started to reduce the amount of S9 produced and used within the industry and for scientific research. Other sources of S9 or metabolizing activity are a possibility, such as human S9, primary human hepatocytes or HepaRG cells (Westerink & Schoonen, 2007a). However, the use of external S9 sources can have a cytotoxic effect and the activity of enzymes can vary greatly depending on the source and S9 lot (Bigger et al., 1980; Kodavanti et al., 2001).

In this study, a protocol proposed by Mollergues et al. (2016) was followed, where S9 was added in a reduced amount and incubated overnight. For Mollergues et al. (2016), the protocol proved to be more efficient for the metabolization of endocrine active substances; however, this was not the case in this study with genotoxic substances, as there was no improved analytical or toxicological sensitivity for the tested substances. The 3 h protocol with increased amounts of S9 on the other hand lead to similar LEC values. Especially for cyclophosphamide, the addition of S9 was crucial, as it would have been negative without it (Figs. S2). For other substances such as BαP no improvements were seen upon S9 addition, leading to the conclusion that the HepG2 cells have a CYP1A1 and CYP1B1 activity, which are necessary for the metabolisation of BαP (Kirkland et al., 2016). Specifically, the viability of BαP with and without S9, as shown in Fig. S3A has to be looked at in more detail. For the protocol with S9 for 3 h, the viability increased to a maximum of 200%. A possible cause for this is the measurement with resazurin, which is metabolized to resorufin. Through the added co-factors and the high concentration of the substance, this can lead to an increase in the metabolic activity of the cells, possibly leading to the increase in viability.

Another important aspect is the activity of detoxifying enzymes, which have to be taken into consideration in the risk assessment (Hakura et al., 2003). This was observed for the substances cisplatin, 2,4-DAT and etoposide, which were positive without S9, but negative with S9 addition, perhaps caused by a detoxification following an activation step, which was also observed in a similar setting by Hughes et al. (2012). This shows that the assay has a good balance in its metabolizing system of (de-)toxifying enzymes. Overall, promising results were obtained without S9 addition for the set of substances tested in this study. However, more substances would need to be analyzed to provide a recommendation whether the use of S9 could be omitted.

For the tested substances the use of an external metabolizing system by adding aroclor 1254 induced rat liver S9 did not lead to a sufficient improvement of sensitivity or specificity, therefore it was concluded that the assay has the potential to work as well without the addition of an external metabolizing system. But, to make a definite recommendation on the use or omission of S9, further experiments would be necessary. For example, without the addition of S9, the substance cyclophosphamide would not have been detected. However, the substance was positive only at very high concentrations, which are well above any relevant concentration where it would appear as an unknown substance in a complex mixture. For complex mixtures, the omission of S9 means that less sample volume would be necessary, which would lead to a reduction in cost and time, which are important for high-throughput screening. Based on our findings so far the testing without S9 is a possibility for an initial pre-screening approach or in a test battery. In general the findings in this study are promising first results, but only apply to the limited amount of substances tested, which were taken from the ECVAM list. To obtain a more comprehensive understanding of the assay’s ability to detect low LEC values, its toxicological sensitivity and specificity and the necessity of an external metabolizing system even more substances would have to be tested. In a guidance document on good *in vitro* method practices the OECD (2018) states that no *in vitro* system can fully mirror the complexity of *in vivo* metabolisms and will always over or underestimate the situation. These considerations should not prevent the use of a metabolizing system or metabolically competent cells, but the limitations of both have to be taken into consideration, as was done here by comparing the addition of an exogenous metabolizing system with that of an endogenous one.

Finally, all these parameters were taken into consideration for the application of complex mixtures, where genotoxic substances might be present in low amounts. Currently used assays are lacking the analytical sensitivity (Rainer et al., 2018; Schilter et al., 2019; Pinter et al., 2020) and this aspect was taken into consideration when developing the assay. Moreover, the applicability and robustness of the assay with complex mixtures was an important aspect during the design of the assay. Further, most genotoxicity assays were developed to analyze pure substances, however, for complex mixtures these assays might have to be re-evaluated (Bopp et al., 2015). With the HepGentox assay in this study a mammalian testing system was developed specifically to analyze complex mixtures and to detect genotoxic substances at lower concentrations. However, this was only done to test complex mixtures deriving from food contact material migrates, to determine whether the assay is applicable also for complex mixtures derived from other sources (such as pharmaceutical impurities, herbal mixtures, or food additives, etc.) the assay would have to be assessed again concerning interference of any matrix effects. Nevertheless, for the analysis of food contact migrates the assay proved to be promising.

As the use of a single mammalian assay is considered to provide insufficient information regarding genotoxicity (Pfuhler et al., 2007; EFSA, 2011), a test battery consisting of more than one assay is commonly applied. The HepGentox assay is no exception and has to be part of a well balanced test battery including other evaluated tests for a comprehensive genotoxicity assessment.

## Conclusions

The HepGentox reporter gene assay showed to be both analytically and toxicologically sensitive to detect a variety of genotoxic substances with different modes of actions. This means that it is able to correctly detect a number of genotoxic substances at low LEC concentrations, which leads to a good analytic sensitivity. Moreover, the high specificity proved that the assay is unlikely to lead to false positive results. Also, the cells showed to have some metabolic activity, so that the omission of S9 is a possibility and it does not have to be included in a first pre-screening approach, but more substances would have to be analyzed to give a recommendation. Since no external metabolism has to be added, the amount of sample required for the test system could be decreased as well, which is often considered a limiting factor. However, it is possible to add S9 at a later stage or when more information is required to verify results of a comprehensive test battery. This makes the assay a good initial tool for genotoxicity testing as it combines several advantageous aspects, such as high-throughput, low sample amount and high sensitivity, all combined in one test system. Therefore, we consider the assay to be a promising candidate for a test battery to test complex mixtures, as it can reliably detect genotoxic substances in the presence of a sample matrix, without any effect towards LEC values or viability. The here presented results show that the assay can provide important information and would be suitable as an initial screening tool as part of a well-balanced test battery for genotoxicity assessment of complex mixture testing.

## Supplemental Information

10.7717/peerj.11883/supp-1 Supplemental Information 1 Viability at different cell concentrations and incubation times A and B show viability measurement of experiments with resazurin after 24 h, with different cell concentrations treated with 4NQO (A) and BαP (B). Diagrams C and D show 2 × 10^4^ cells/well treated with 4NQO (C) and BαP (D) for 6, 24, 48 and 72 h. *X*-axis show the concentration of the genotoxic substances and *y*-axis the viability, which was compared to the background as benchmark of 100% viability. The dashed line indicates the threshold of 70% viability, below which it is regarded as cytotoxic. The data show the mean of at least three independent experiments with twelve replicates each.

10.7717/peerj.11883/supp-2 Supplemental Information 2 Nluc of pure substances tested with varying S9 concentration The diagrams show Nluc measurement of experiments with HepGentox cells treated with different substances: 2-AAF (A), aflatoxin B1 (B), BαP (C), cisplatin (D), cyclophosphamide (E), 2,4-DAT (F), ENU (G), etoposide (H). *X*-axis show the concentration of the genotoxic substances and *y*-axis the fold induction, which was calculated with the mean Nluc value divided by the mean background (1% DMSO). The dashed line indicates the threshold of 1.7 (background + 3 times standard deviation), above which the first signal was taken as LEC value. The data show the mean of at least three independent experiments with twelve replicates each.

10.7717/peerj.11883/supp-3 Supplemental Information 3 Viability of pure substances tested with varying S9 concentration The diagrams show the viability measurement of experiments with HepGentox cells treated with different substances: 2-AAF (A), aflatoxin B1 (B), BαP (C), cisplatin (D), cyclophosphamide (E), 2,4-DAT (F), ENU (G), etoposide (H). *X*-axis show the concentration of the genotoxic substances and *y*-axis the viability, which was compared to the background as benchmark of 100% viability. The dashed line indicates the threshold of 70% viability, below which it is regarded as cytotoxic. The data show the mean of at least three independent experiments with twelve replicates each.

10.7717/peerj.11883/supp-4 Supplemental Information 4 Comparison of the HepGentox assay to the mammalian reporter gene assays the BlueScreen HCTM and the p53 CALUX^®^ (+): value obtained with S9 addition (-): value obtained without S9 N/A: no LEC data was found in the literature for a substance with the respective assay 1(Hughes et al., 2012), 2(Van der Linden et al., 2014))

10.7717/peerj.11883/supp-5 Supplemental Information 5 Raw Data for Fig. 1

10.7717/peerj.11883/supp-6 Supplemental Information 6 Raw Data for Fig. 2

10.7717/peerj.11883/supp-7 Supplemental Information 7 Raw Data for Fig. 3

## Additional Information and Declarations

### Data Availability

The following information was supplied regarding data availability: The raw data for the experiments are available in the Supplemental Files.
