# Supplementary figures and images for: HepGentox: a novel promising HepG2 reportergene-assay for the detection of genotoxic substances in complex mixtures
Source: PeerJ. 2021 Jul 27;9:e11883. doi: 10.7717/peerj.11883 (PMC8323594; doi:10.7717/peerj.11883)

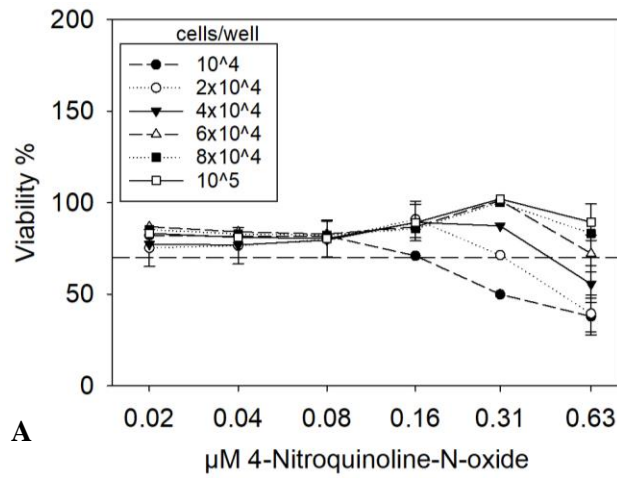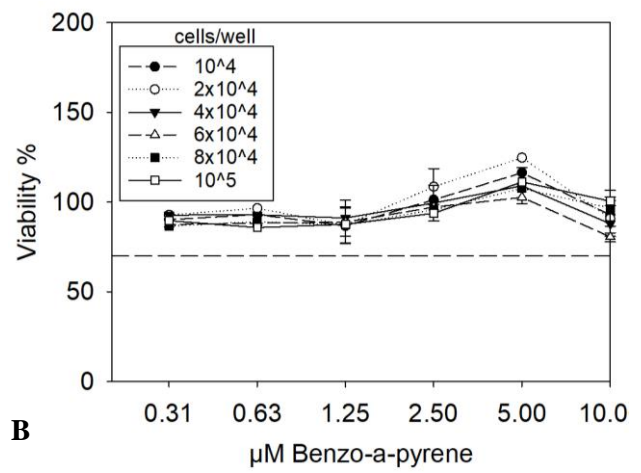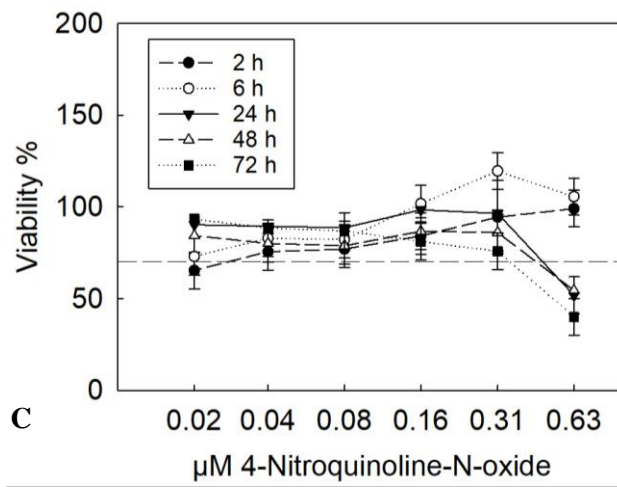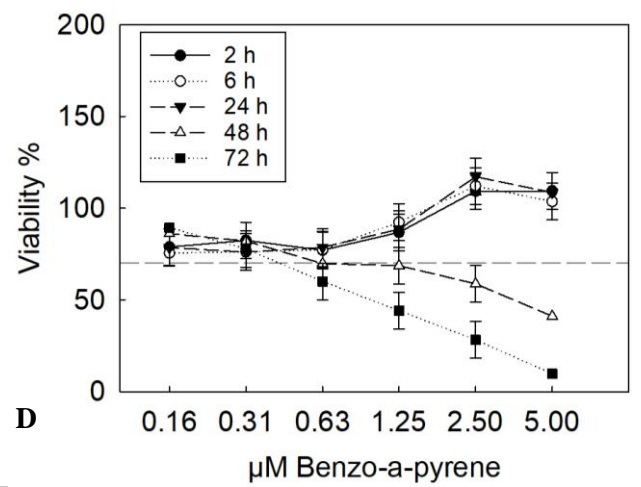

Supplement: Supplemental Information 1 — A and B show viability measurement of experiments with resazurin after 24 h, with different cell concentrations treated with 4NQO (A) and BαP (B). Diagrams C and D show 2 × 10^4^ cells/well treated with 4NQO (C) and BαP (D) for 6, 24, 48 and 72 h. X-axis show the concentration of the genotoxic substances and y-axis the viability, which was compared to the background as benchmark of 100% viability. The dashed line indicates the threshold of 70% viability, below which it is regarded as cytotoxic. The data show the mean of at least three independent experiments with twelve replicates each. [file peerj-09-11883-s001.pdf]

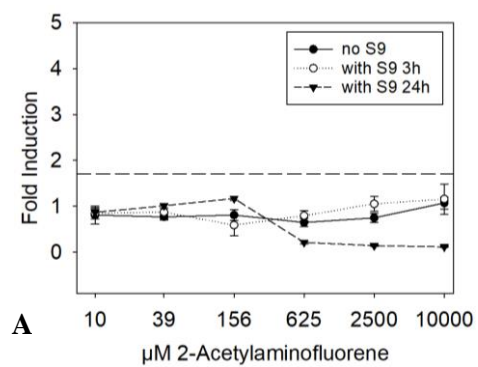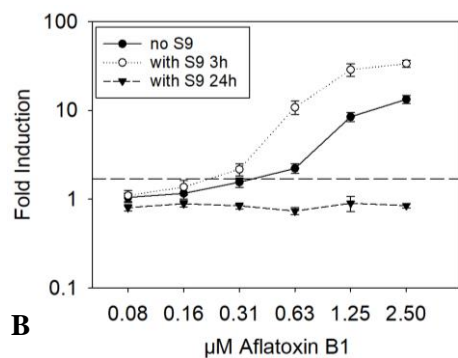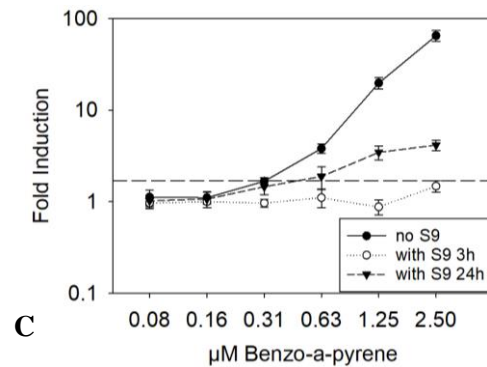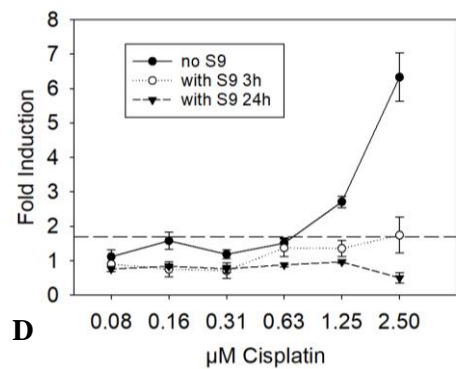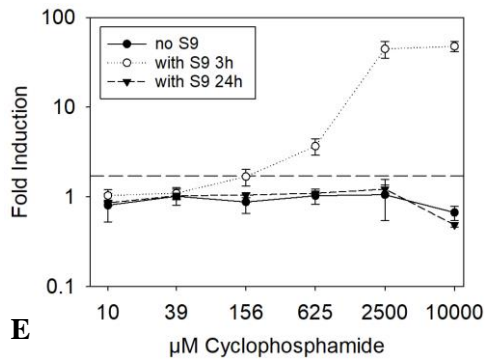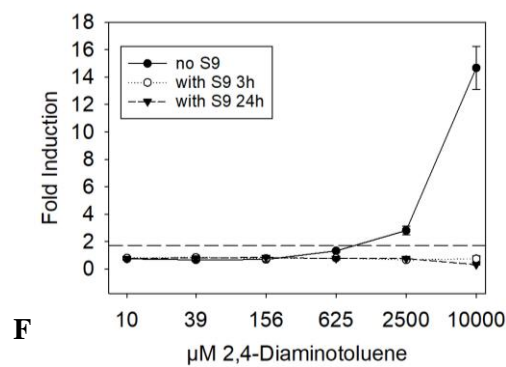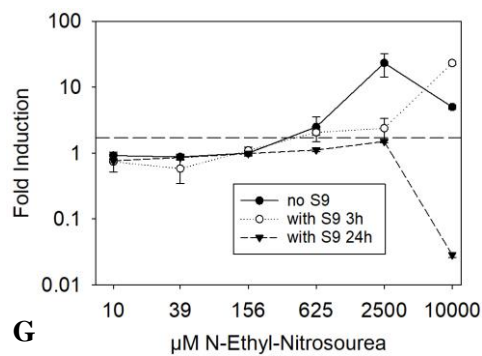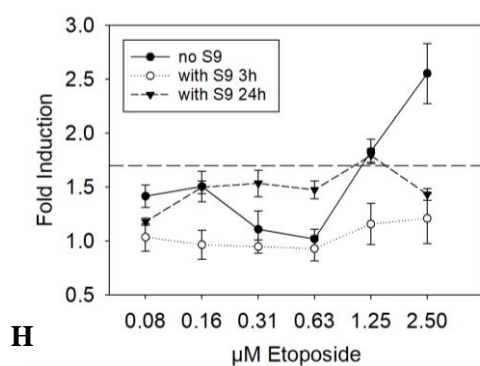

Supplement: Supplemental Information 2 — The diagrams show Nluc measurement of experiments with HepGentox cells treated with different substances: 2-AAF (A), aflatoxin B1 (B), BαP (C), cisplatin (D), cyclophosphamide (E), 2,4-DAT (F), ENU (G), etoposide (H). X-axis show the concentration of the genotoxic substances and y-axis the fold induction, which was calculated with the mean Nluc value divided by the mean background (1% DMSO). The dashed line indicates the threshold of 1.7 (background + 3 times standard deviation), above which the first signal was taken as LEC value. The data show the mean of at least three independent experiments with twelve replicates each. [file peerj-09-11883-s002.pdf]

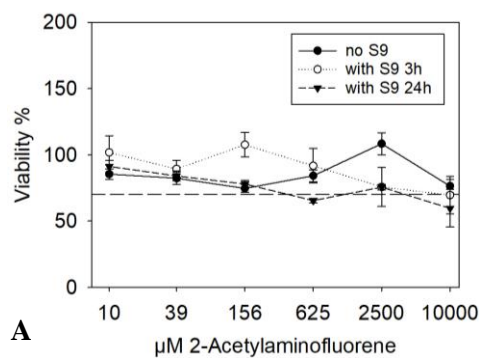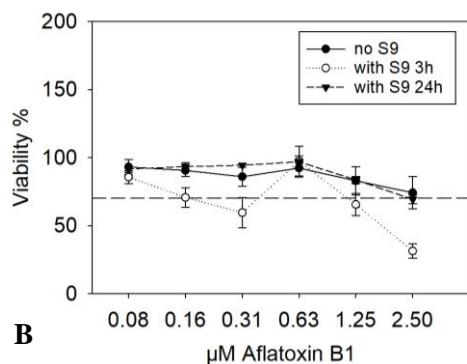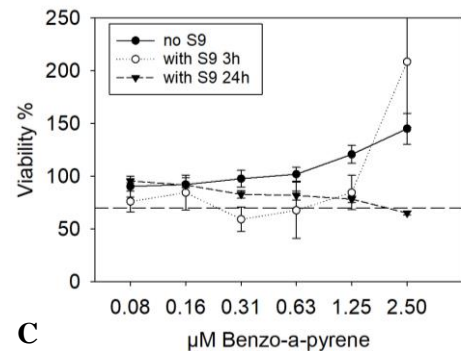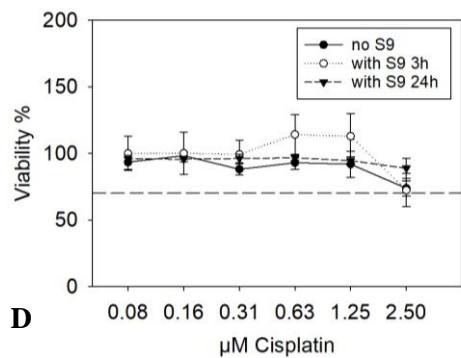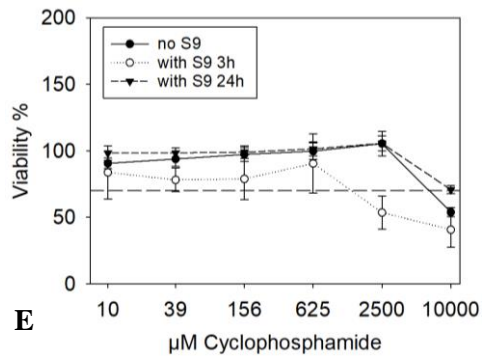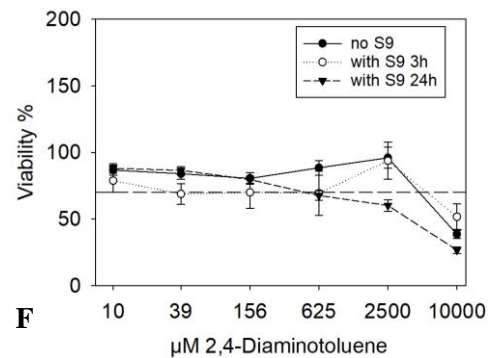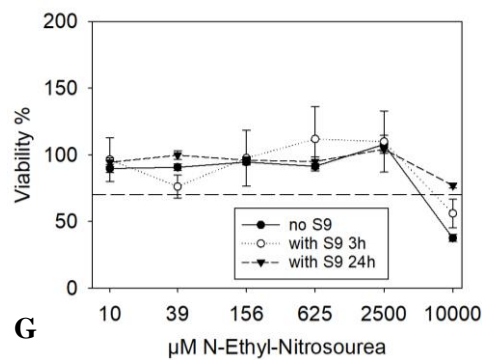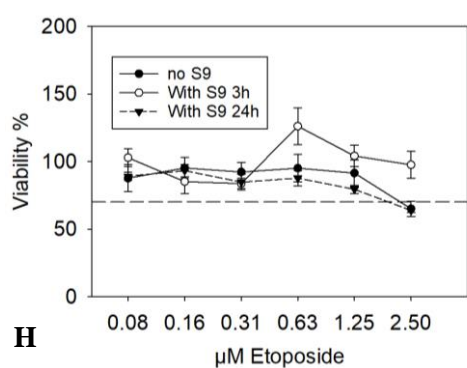

Supplement: Supplemental Information 3 — The diagrams show the viability measurement of experiments with HepGentox cells treated with different substances: 2-AAF (A), aflatoxin B1 (B), BαP (C), cisplatin (D), cyclophosphamide (E), 2,4-DAT (F), ENU (G), etoposide (H). X-axis show the concentration of the genotoxic substances and y-axis the viability, which was compared to the background as benchmark of 100% viability. The dashed line indicates the threshold of 70% viability, below which it is regarded as cytotoxic. The data show the mean of at least three independent experiments with twelve replicates each. [file peerj-09-11883-s003.pdf]
