# Supplementary material for: HepGentox: a novel promising HepG2 reportergene-assay for the detection of genotoxic substances in complex mixtures
Source: PeerJ. 2021 Jul 27;9:e11883. doi: 10.7717/peerj.11883 (PMC8323594; doi:10.7717/peerj.11883)
Supplement: Supplemental Information 4 — (+): value obtained with S9 addition (-): value obtained without S9 N/A: no LEC data was found in the literature for a substance with the respective assay 1(Hughes et al., 2012), 2(Van der Linden et al., 2014)) [file peerj-09-11883-s004.pdf]

Substance	CAS	HepGentox [μg/mL]	BlueScreen HC™ [μg/mL][1]	P53 CALUX® [μg/mL][2]
Cyclophosphamide	6055-19-2	88	6.25 (+)	279 (+)
N-Ethyl-nitrosourea	759-73-9	73	37.5 (-)	117 (-)
Methyl methanosulfonate	66-27-3	69	12.5 (-)	33 (-)
Benzo-a-pyrene	50-32-8	0.2	2.5 (+)	2.5 (+)
7,12-Dimethylbenzanthracene	57-97-6	0.4	5 (+)	0.8 (+)
2-Acetylaminofluorene	53-96-3	Negative	200 (+)	2 (-)
2,4-Diaminotoluene	95-80-7	76	125 (-)	122 (+)
Aflatoxin B1	1162-65-8	0.2	0.08 (+)	0.9 (+)
Cisplatin	15663-27-1	0.2	1.0 (-)	3 (+)
Sodium arsenite	7784-46-5	8	0.63 (-)-	0.013 (-)
Etoposide	33419-45-0	0.8	0.13 (-)	6 (-)
4-Nitroquinoline-n-oxide	56-57-5	0.03	0.13 (-)	N/A
Colchicine	64-86-8	Negative	N/A	N/A
Mitomycin C	50-07-7	0.1	N/A	N/A
Actinomycin D	50-76-0	1.6	N/A	0.013 (-)
Doxorubicin	23214-92-8	0.03	N/A	N/A
